# The inequity of education, health and care plan provision for children and young people with intellectual and developmental disabilities

**DOI:** 10.1111/jir.13139

**Published:** 2024-05-22

**Authors:** I. O. Lee, J. Wolstencroft, H. Housby, M. B. M. van den Bree, S. J. R. A. Chawner, J. Hall, IMAGINE ID Consortium, D. H. Skuse

**Affiliations:** ^1^ Behavioural and Brain Sciences Unit, Population Policy and Practice Programme, Great Ormond Street Institute of Child Health University College London London UK; ^2^ Medical Research Council Centre for Neuropsychiatric Genetics and Genomics, Division of Psychological Medicine and Clinical Neurosciences Cardiff University Cardiff UK

**Keywords:** education, health and care plan, index of multiple deprivation, inequity, intellectual and developmental disability, regions of England, socioeconomic status

## Abstract

**Background:**

Children and young people (CYP) with intellectual and developmental disabilities (IDDs) have significant additional educational needs compared with the general population. In England, the government has established a system of education, health and care plans (EHCPs) to support children with special educational needs and disabilities, but disparities exist between the degree of need and the availability of support. We conducted a prospective UK national cohort study (IMAGINE) of children with rare pathogenic genomic variants, all of which are associated with IDD, to investigate associated neuropsychiatric risk. Subsequently, we obtained information from the UK's National Pupil Database on their educational progress through the state school system. We aimed to identify whether they had received EHCP provision and whether that support was associated with their family's socioeconomic status, region of domicile, ethnicity, sex, primary special educational needs (SEN) type, academic performance and mental health well‐being.

**Methods:**

We recruited 2738 CYP from England into the IMAGINE study between 2014 and 2019. The educational histories of the participants (6–28 years old, mean ± standard deviation = 14 ± 4 years, 56% male) were obtained from the Department for Education's National Pupil Database in 2021. Educational data included attainment scores from the Early Year Foundation Stage (<5 years) to key stage 4 (15–16 years). Each family was assigned an index of multiple deprivation (IMD) score based on their home address postcode. Parents or carers rated their child's emotional and behavioural adjustment on the Strengths and Difficulties Questionnaire (SDQ). The association between receiving an EHCP and the child's IMD score, eligibility for free school meals, English region of domicile, ethnicity, sex, primary SEN type, academic attainment and SDQ score was investigated.

**Results:**

In this cohort, 78% of participants had received an EHCP. CYP living in the most deprived IMD deciles were substantially less likely to receive EHCP support than those in the least deprived decile, irrespective of their degree of intellectual developmental disability, academic performance or associated mental health problems. There were no sex differences. Children of Asian heritage were more likely to have been granted an EHCP than White children from equivalent IMD deciles. There were striking regional disparities. Participants living in London were significantly more likely to have been awarded an EHCP than participants living anywhere else in England, regardless of their IMD decile; those in the least deprived decile had almost 100% EHCP provision.

**Conclusions:**

This study found evidence for nationwide regional inconsistencies in the awarding of EHCP to CYP with significant intellectual impairments of known genetic aetiology. Disparities in funds available to education authorities could be a contributory factor. EHCP support was potentially influenced by how strongly a parent advocates for their child.

**Abbreviations**
CI: confidence interval
CYP: children and young people
DfE: Department for Education
EHCP: education, health and care plan
EYFSP: Early Years Foundation Stage Profile
FSM: free school meal
IDD: intellectual and developmental disability
IMAGINE: Intellectual Disability and Mental Health: Assessing the Genomic Impact on Neurodevelopment
IMD: index of multiple deprivation
KS: key stage
NPD: National Pupil Database
ONS: Office for National Statistics
OR: odds ratio
PHI: equivalent to correlation coefficient
SD: standard deviation
SDQ: Strengths and Difficulties Questionnaire
SEN: special educational needs
SEND: special educational needs and disabilities

## Introduction

Children and young people (CYP) with intellectual and developmental disabilities (IDDs) have substantial additional educational needs compared with typically developing pupils. IDD affects 1.7% of the population worldwide (Nair *et al*. 2022) and is an important socioeconomic issue in healthcare and education (Ilyas *et al*. 2020; Mon‐Williams & Wood 2023). It has been associated with increased mortality and morbidity, increased risk of social exclusion, and significant demands on families and health and social care providers (Einfeld & Emerson 2008; Emerson 2012).

In England, about 1.5 million pupils are recognised to have special educational needs and disabilities (SEND) (UK Government 2023a). Educational support to CYP in schools varies according to their special educational needs (SEN) status (UK Government 2023b). Two levels of provision are in place in England. At the first level is SEN support, previously called School Action (flagging that a child requires help but not necessarily providing any) or School Action Plus (UK Government 2022a), which is a school‐specific learning programme. Neither is associated with additional school funding. At the second level is an education, health and care plan (EHCP), which replaces the previous SEN statement and was introduced as part of the Children and Families Act 2014 (UK Government 2021). An EHCP identifies the child's educational, health and social needs and sets out the additional support required to meet those needs. It instructs educational authorities on what additional resources are required for that child's education, which could include speech and language therapy, physiotherapy, occupational therapy and clinical psychotherapy (UK Government 2023b). In 2023, 13% of pupils in state‐funded schools have SEN support, and 4% have been awarded an EHCP. Both proportions have increased since 2016 (UK Government 2023a), placing an additional demand on education authority finances at a time when there has been a 9% real‐term fall in school funding since 2010.

It is well recognised that EHCPs are not provided to all pupils with substantial educational needs (Richards 2022). There are disparities associated with ethnicity and sex differences (House of Commons, U.K. 2020). Regional differences in the proportion of eligible SEND pupils who have been awarded an EHCP have been described as a ‘postcode lottery’ (House of Commons, U.K. 2020). Children from more disadvantaged socioeconomic backgrounds are at increased risk of requiring additional educational support (Croll 2002; Wood *et al*. 2024), but are less likely to get educational support than those in more affluent areas (Hutchinson *et al*. 2020). Ideally, EHCP provision to children with significant IDD should be made soon after school entry; delays can result in negative outcomes, including disengagement, exclusion, poor academic and health progress with consequent long‐term physical and mental health problems (Emerson 2012; Parker *et al*. 2016; Lőrinc *et al*. 2020).

In this study, we aimed to investigate the equity of EHCP provision to a cohort of CYP with IDD of known genetic aetiology in a nationwide survey. We assessed variations in provision with regard to socioeconomic status [index of multiple deprivation (IMD) and free school meal (FSM) eligibility], region of domicile, ethnicity, sex, primary SEN type, academic performance and mental health status. We hypothesised that all these factors could affect whether an EHCP would be granted.

## Material and methods

### Study participants

This cohort study follows on from the previous project, titled the Intellectual Disability and Mental Health: Assessing the Genomic Impact on Neurodevelopment (IMAGINE) study, which originally recruited 3407 UK participants between 2014 and 2019 (Wolstencroft *et al*. 2022). To be eligible, participants were required to be aged at least 4 years at the time of enrolment, to have a developmental delay or an intellectual disability diagnosis made by a clinical care team and to have a confirmed molecular genetic diagnosis documented from an accredited diagnostic laboratory. Recruitment to the study was by referral from 23 UK regional genetics centres (76%) and self‐referrals or patient support groups (24%). A parent or guardian provided consent on behalf of children younger than 16 years. For individuals older than 16 years who did not have capacity, consultees acted on their behalf. The study was approved by the London Queen Square Research Ethics Committee (14/LO/1069).

This present study focused on a subset of 2738 individuals with genetic anomalies aged 6–28 years [mean ± standard deviation (SD) = 14.8 ± 4.4; 56% male]. We obtained data on their educational histories from the National Pupil Database (NPD) for England (Scotland, Wales and Northern Ireland have other record systems). Participants in IMAGINE were more likely to be of White ethnic origin (89% in Table 1), compared with the general population (81.0%) [Office for National Statistics (ONS) 2022]. Asian (e.g. Pakistani, Indian origins, Chinese and others, 4.3%) and Black (0.9%) children were under‐represented compared with the general population (9.6% Asian and 4.6% Black). We lack information on who was approached to participate by their regional genetic centres, so we do not know whether this disparity implies fewer children from those ethnic groups were sent for genetic screening or whether they were relatively less likely to consent to participate than the White participants.

**Table 1:** Participant demographic information

Demographic category	Frequency, *N* (% of total count)	95% CI Cohort proportion	National percentage in England (Office for National Statistics 2022)	
Total	2738			
Sex			
Male	1535 (56%)	54.2–57.9%	49%	
Female	1203 (44%)	42.1–45.8%	51%	
Age at recruitment (years)			
Mean ± SD [95% CI]	13.8 ± 4.3	[13.7–14.0]		
Median	13.3			
Range	6.4–27.7			
Age group at recruitment (years)			
6–7	161 (5.9%)	5.0–6.76%		
8–11	910 (33.2%)	31.4–35.0%	
12–14	690 (25.2%)	23.6–26.8%		
15–16	348 (12.7%)	11.5–13.9%		
17–18	260 (9.5%)	8.4–10.6%		
Post‐19	369 (13.5%)	12.2–14.8%		
Ethnicity			
White	2445 (89.3%)	88.1–90.5%	81.0%	
Asian	119 (4.3%)	3.6–5.1%	9.6%	
Black	24 (0.9%)	0.5–2.3%	4.2%	
Mixed	132 (4.8%)	4.0–5.6%	3.0%	
Any other ethnic group	18 (0.7%)	0.4–0.9%	2.2%	
Primary SEN type^†^			
PMLD	226 (8.3%)	7.3–9.3%	0.8%	
Severe LD	625 (22.8%)	21.2–24.4%	2.3%	
Moderate LD	437 (16.0%)	14.6–17.4%	15.3%	
Specific LD	208 (7.6%)	6.6–8.6%	11.7%	
SLCN	474 (17.3%)	15.9–18.7%	23.7%	
ASD	461 (16.8%)	15.4–18.2%	14.2%	
SEMH + BESD	93 (3.4%)	2.7–4.1%	19.6%	
MSI + HI + VI	29 (1.1%)	0.7–1.5%	2.6%	
PD	71 (2.6%)	2–3.2%	2.5%	
Other difficulties/disabilities	65 (2.4%)	1.8–3.0%	3.6%	
Missing data	49 (1.8%)	1.3–2.3%	3.4%	
**Index of multiple deprivation decile**			**National %**	**% aged under 25** ^‡^
1 (most deprived)	303 (11.1%)	9.9–12.3%	9.9%	12.0%
2	282 (10.3%)	9.2–11.4%	10.1%	11.4%
3	269 (9.8%)	8.7–10.9%	10.3%	11.0%
4	253 (9.2%)	8.1–10.3%	10.2%	10.3%
5	276 (10.1%)	9.0–11.2%	10.1%	9.8%
6	263 (9.6%)	8.5–10.7%	10.2%	9.6%
7	247 (9.0%)	7.9–10.1%	9.9%	9.1%
8	273 (10%)	8.8–11.1%	9.9%	9.0%
9	274 (10%)	8.9–11.1%	9.8%	8.9%
10 (least deprived)	298 (10.9%)	9.7–12.1%	9.7%	9.1%
Free school meal eligibility				
No	1774 (64.8%)	63–66.6%	77.5%	
Yes	964 (35.2%)	33.4–37.0%	22.5%	
Regions of England				
North East	109 (4.0%)	3.2–4.7%	4.7%	
North West	247 (9.0%)	7.9–10.1%	13.0%	
Yorkshire and the Humber	394 (14.4%)	13.1–15.7%	9.8%	
East Midlands	192 (7.0%)	6.0–8.0%	8.6%	
West Midlands	368 (13.4%)	12.2–14.7%	10.5%	
East of England	423 (15.4%)	14.1–16.8%	11.1%	
London	266 (9.7%)	8.6–10.8%	15.9%	
South East	531 (19.4%)	17.9–21%	16.3%	
South West	208 (7.6%)	6.6–8.6%	10.0%	

^†^ The secondary SEN types are represented in Table S1.

^‡^ EHCP could be granted by the DfE up to the age of 25.

ASD, autistic spectrum disorder; BESD, behavioural, emotional and social difficulty; CI, confidence interval; EHCP, education, health and care plan; HI, hearing impairment; LD, learning difficulty; MSI, multi‐sensory impairment; *N*, number of cases; PD, physical disability; PMLD, profound and multiple learning disabilities; Post‐19, 19 years old onward (i.e. 19–27.7 in this cohort); SD, standard deviation; SEMH, social, emotional and mental health; SEN, special educational needs; SLCN, speech, language and communication needs; VI, visual impairment.

### Data source and description

All participants were educated in English mainstream or special educational schools at some point between 2006 and 2021. Personal data were linked to their educational histories derived from the NPD, which was managed by the UK Department for Education (DfE). The NPD provided additional details on ethnicity, socioeconomic impoverishment (indicated by FSM eligibility) and primary SEN type. Our cohort with IDD had substantially higher percentages of participants with primary SEN types profound and multiple learning disabilities (PMLD) and severe learning difficulty (LD) compared with that of the national SEND pupils across England (see Table 1 and secondary SEN type in Table S1). Postcodes were used to calculate the IMD deciles (1 = most deprived; 10 = least deprived). In the current English Indices of Deprivation 2019, seven domains of deprivation are considered and weighted as follows: income (22.5%), employment (22.5%), education (13.5%), health (13.5%), crime (9.3%), barriers to housing and services (9.3%) and living environment (9.3%). The proportions of the cohort within each decile of the IMD distribution were similar both to the general population of England and to those aged under 25 (Table 1). Home addresses were categorised into nine regions of England according to the UK ONS.

Primary caregivers completed the Strengths and Difficulties Questionnaire (SDQ) (Goodman 1997), which is a globally recognised instrument for assessing CYP's emotional and behavioural adjustment in dimensional terms that reflect their mental health state (Goodman 2001). It has been used as a standardised assessment to evaluate emotional and behavioural difficulties in CYP with IDDs (Murray *et al*. 2020). The SDQ comprises four problem domain sub‐scales: emotional symptoms; conduct problems; hyperactivity, impulsivity and inattention difficulties; and peer relationship problems. There is also a scale for prosocial behaviour. Problem scales are combined to yield a total difficulties score (Goodman 2001; Goodman & Goodman 2009). The SDQ scores were categorised into four bands based on general population survey data (see additional information in Note S1): 80% of UK children score in a ‘close to average’ range regarded as ‘normal’, 10% score in a ‘slightly raised’ range as ‘borderline’ and 10% score in the ‘high’ or ‘very high’ range, indicative of potential clinical significance (Terapia 2020).

The NPD records provided information about school types (special school, mainstream school or pupil referral unit), whether the pupil had been in the SEN unit of a mainstream school and whether they had been granted an EHCP or SEN statement (term used before 2014; Note S2). Herein, the term ‘EHCP’ will be used to denote the acquisition of an EHCP or SEN statement, which entitled the student to a higher level of support. A variable named ‘Age at first record of obtaining an EHCP’ (in years) was calculated between the year when the pupil first received an EHCP and the year of birth.

The education attainment data provided by the DfE were based on the academic performance of the pupils as measured by standardised tests at different stages of their school careers. Table 2 provides information on the age at which pupils are assessed at each educational stage, the measures made of academic achievement, and the national average or expected scores. The number of pupils for whom data were provided by the DfE is also shown; the proportions at each educational stage decrease over time because fewer SEND pupils were capable of being tested beyond that point. The Early Years Foundation Stage Profile (EYFSP) measures five main learning areas at 4–5 years: a phonics mark assesses reading and writing skills at ages 5–6; key stage 1 (KS1) assesses reading, writing, mathematics and overall science at ages 6–7; key stage 2 (KS2) assesses attainments in reading, writing and mathematics at ages 10–11; at key stage 3 (KS3), attainments in English, mathematics and science are assessed at age 14; and key stage 4 (KS4) comprises a national examination in a number of different subjects, which is graded. A pass indicates attainments of at least grade C, and the expected level for a typical pupil would be at least five passes (UK DfE 2016; UK Government 2022b). Only 18.4% (426) of pupils for whom we have data from the DfE attempted KS4.

**Table 2:** Statutory assessment (stage), age at assessment, measurement domain, number of participants with relevant data, and the national average or expected of the scores for each stage

Statutory assessment	Age (years)	Measurement domain	Number of participants with DfE data	National average/expected level
Early Years Foundation Stage Profile (EYFSP) score	4–5	Personal, social and emotional development; communication, language and literacy; mathematical development; knowledge and understanding of the world; physical development; and creative development	2312	34.6
Phonics mark	5–6	Reading and writing	1217	32
Key stage 1 (KS1) average scaled attainment score	6–7	Reading, writing, mathematics and overall science	1209	100
Key stage 2 (KS2) average scaled attainment score	10–11	Reading, writing and mathematics	906	100
Key stage 3 (KS3) teacher assessment	14	English, mathematics and science	129	Level 5
Key stage 4 (KS4) General Certificate of Secondary Education (GCSE) examination grade	16	Various GCSE subjects	426	Pass = 5 subjects and above reach GCSE grades A*–C

### Statistical analysis

Chi‐squared (*χ* ^2^) tests were used to compare proportions of participants with and without an EHCP when the cohort was stratified by socioeconomic status (IMD decile and FSM eligibility), region of domicile, ethnicity and sex. Mann–Whitney *U*‐tests or *t*‐tests were used to compare statutory academic attainment scores and SDQ scores. A stepwise logistic regression model assessed whether or not the child had been granted an EHCP (dependent variable) within each IMD decile (independent variable) when covariating variables: region of England, FSM eligibility, ethnicity and sex. In all analyses, a *P*‐value <0.05 indicated acceptable statistical significance. Analyses were performed in spss (version 24) on the ONS Secure Research Service platform. Statistical disclosure guidelines from the ONS forbid counts less than 10 from being published, for data protection purposes. Therefore, exact numbers for certain minority groups (e.g. ethnic) cannot be presented in this article. Suppression for the extended data has also been performed accordingly.

## Results

### Age, sex and school types

Overall, 77.8% of participants had received an EHCP (*N* = 2131/2738) at the point of their participation in our study. The mean age at which an EHCP was awarded was 7.1 ± 2.6 years (mean ± SD, median = 7.2); the mean age of those who did not have an EHCP was 12.9 ± 4.0 years old at the time of their study participation (median = 12.5 years old). The sex distribution was similar in both groups: 57% and 54% were male, respectively (*χ* ^2^ = 1.52, *P* = 0.22). There were no significant sex differences in the age of receiving an EHCP (*t* = 0.49, *P* = 0.626).

Of those with an EHCP in this cohort, about one‐third (35%, *N* = 613/1761) were being educated in special schools for children with learning disabilities at the time of their KS1. The remaining (65%, *N* = 1148/1761) were attending mainstream schools (including community, academy and voluntary‐aided schools). The proportion of pupils studying in special schools was greater at subsequent stages: 52% (*N* = 548/1057) at KS2, 73% (*N* = 74/101) at KS3 and 75% (*N* = 290/388) at KS4; 8.2% (*N* = 75/915) had attended a mainstream school with a SEN unit.

For those without an EHCP (*N* = 607), almost all cohort pupils were educated in mainstream schools at all key stages: KS1 (*N* = 483/483), KS2 (244/245), KS3 (18/18) and KS4 (58/60), and less than 10 pupils were in a pupil referral unit at KS2 and KS4. Less than 10 participants (<1%) were reported to have attended a SEN unit within mainstream schools. None of those without an EHCP had attended a special school, reflecting the fact that an EHCP is required in order to receive specialised education.

### Primary special educational needs type

The cohort participants with IDD had multiple primary SEN types recorded throughout their educational histories. The primary SEN types described in Table 3 were selected according to their most frequently recorded status. Over 94% of participants within each of the two SEN types associated with the most significant learning disabilities, PMLD and severe LD, had received an EHCP. Within the other primary SEN types, the percentages of participants receiving EHCP ranged from 46.2% (other difficulties/disabilities) to 79.6% (autistic spectrum disorder).

**Table 3:** Comparison of the cohort participants with and without EHCPs by the primary SEN types

Primary SEN type	No EHCP		Have EHCP	
	Frequency (% of subgroup)	95% CI	Frequency (% of subgroup)	95% CI
PMLD	<10		<226 (>94%)	
Severe LD	<10		<625 (>98%)	
Moderate LD	115 (26.3%)	24.7–27.9%	333 (73.7%)	72.1–75.3%
Specific LD	79 (38.0%)	36.2–39.8%	129 (62.0%)	60.2–63.8%
SLCN	136 (28.7%)	27.0–30.4%	338 (71.3%)	69.6–73.0%
ASD	94 (20.4%)	18.9–21.9%	367 (79.6%)	78.1–81.1%
SEMH + BESD	47 (50.5%)	48.6–52.4%	46 (49.5%)	47.6–51.4%
MSI + HI + VI	13 (44.8%)	42.9–46.7%	16 (55.2%)	53.3–57.1%
PD	28 (39.4%)	37.6–41.2%	43 (60.6%)	58.8–62.4%
Others	35 (53.8%)	51.9–55.7%	30 (46.2%)	44.3–48.1%

<10, count less than 10 cannot be presented according to the Office for National Statistics guidelines; ASD, autistic spectrum disorder; BESD, behavioural, emotional and social difficulty; CI, confidence interval; EHCP, education, health and care plan; HI, hearing impairment; LD, learning difficulty; MSI, multi‐sensory impairment; Others, other difficulties/disabilities; PD, physical disability; PMLD, profound and multiple learning disabilities; SEMH, social, emotional and mental health; SEN, special educational needs; SLCN, speech, language and communication needs; VI, visual impairment.

### Academic performance differences

**Figure 1:**
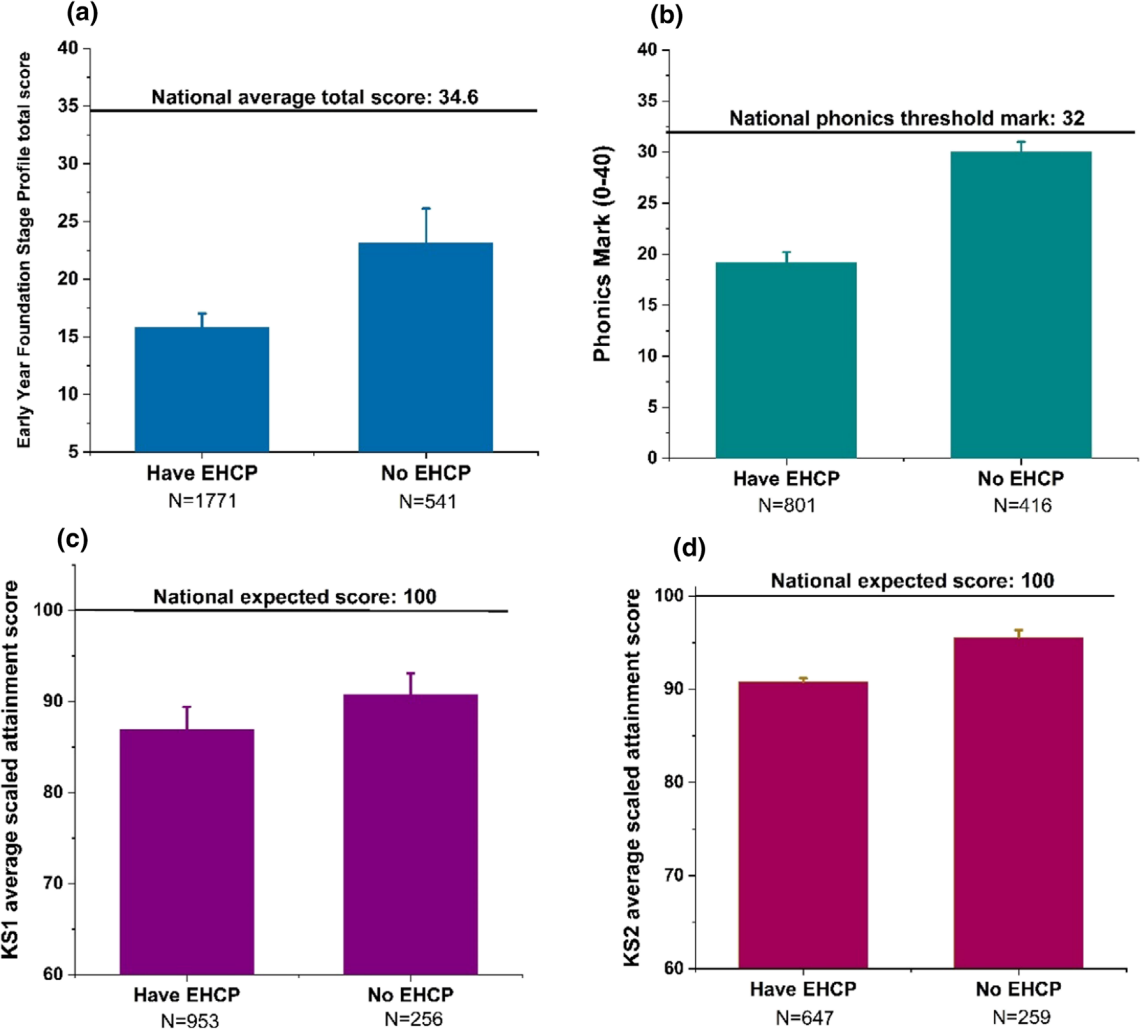
Academic performance of the participants with and without education, health and care plan (EHCP) at four educational stages. (a) The mean Early Years Foundation Stage Profile (EYFSP) total scores. (b) The phonics marks. (c) The key stage 1 (KS1) average scaled attainment scores. (d) The KS2 average scaled attainment scores. The mean and 95% confidence interval error bars are shown. Horizontal lines represent the scores required to meet the national standard at each educational stage.

We investigated whether pupils without an EHCP had achieved the national attainment standard at the six stages of academic attainment {i.e. EYFSP, phonics, KS1, KS2, KS3 and KS4 [General Certificate of Secondary Education (GCSE)]}. We found that mean scores at EYFSP, phonics, KS1 and KS2 were below the national expected scores for both those with and without an EHCP. Both groups scored similarly at the EYFSP (*P* = 0.357); those who had been granted an EHCP obtained significantly lower scores than those without an EHCP, indicating poorer academic progress (all *P*‐values <0.001; Fig. 1a–d). At each national assessment timepoint, children with and without an EHCP performed less well, on average, than the expected national threshold for their age (Fig. 1). At KS3, we only received academic data for 129 cohort pupils (of whom 19.4% did not have an EHCP). At KS4, 34% of pupils without an EHCP achieved at least one GCSE pass, compared with 77 pupils (11%) in the group with an EHCP. The low numbers of pupils that attended GCSE examinations in both groups reflect the fact that, because of their IDD, most could not appropriately be entered at 16 years.

### Emotional and behavioural difficulties – Strengths and Difficulties Questionnaire scores

Table 4 shows the total and sub‐scale SDQ scores, which have been categorised into bands (bracketed) according to UK population norms. Comparing the parent‐rated emotional and behavioural difficulties of those with and without an EHCP, those without an EHCP had significantly higher emotional symptom scores (*t* = 6.58, *P* < 0.001) and conduct problem scores (*t* = 5.95, *P* < 0.001) than those with an EHCP, indicating they were perceived to have greater emotional difficulties and conduct problems. Those with an EHCP had significantly lower prosocial behaviour scores than those without an EHCP (*t* = −7.87, *P* < 0.001).

**Table 4:** Strengths and Difficulties Questionnaire scores of the participants with and without EHCP

SDQ score mean ± SD [95% CI] (classified band)	No EHCP	Have EHCP	UK norm (80% population)
*N*	510	1857	
Total difficulties	21.5 ± 7 [20.83–22.11] (very high)	19.9 ± 7 [19.6–20.2] (very high)	0–13 (average)
Emotional symptoms	5.4 ± 3 [5.1–5.6] (high)	4.4 ± 3 [4.3–4.5] (slightly raised)	0–3 (average)
Conduct problems	4.0 ± 3 [3.8–4.3] (high)	3.3 ± 2 [3.1–3.4] (slightly raised)	0–2 (average)
Hyperactivity	7.5 ± 2 [7.3–7.8] (slightly raised)	7.8 ± 2 [7.7–7.9] (slightly raised)	0–5 (average)
Peer problems	4.5 ± 2 [4.3–4.8] (high)	4.5 ± 2 [4.4–4.6] (high)	0–2 (average)
Prosocial behaviour	6.0 ± 3 [5.8–6.3] (low)	5.0 ± 3 [4.9–5.1] (very low)	8–10 (average)

‘Very high’ and ‘high’ indicate abnormally high difficulties compared with the general population. ‘Slightly raised’ indicates borderline difficulties between normal and abnormal. ‘Low’ and ‘very low’ indicate abnormally low abilities compared with the general populations.

CI, confidence interval; EHCP, education, health and care plan; SD, standard deviation; SDQ, Strengths and Difficulties Questionnaire.

### Index of multiple deprivation decile

Within the country as a whole, pupils living in more deprived IMD deciles were less likely to have been granted an EHCP than pupils from more advantaged IMD deciles (*χ* ^2^ = 77.4, *P* < 0.001; Table 5).

**Table 5:** Comparison of participants with and without EHCPs by IMD decile, region of England, free school meal eligibility and ethnicity

Variable	No EHCP		Have EHCP		*χ* ^2^	PHI	*P*‐value
	Frequency (% of subgroup)	95% CI	Frequency (% of subgroup)	95% CI			
IMD decile					77.4	0.168	<0.001
1	112 (37.0%)	35.2–38.8%	191 (63.0%)	61.2–64.8%			
2	78 (27.7%)	26.0–29.4%	204 (72.3%)	70.6–74.0%			
3	60 (22.3%)	20.7–23.9%	209 (77.7%)	76.1–79.3%			
4	69 (27.3%)	25.6–29.0%	187 (72.7%)	71.0–74.4%			
5	67 (24.3%)	22.7–25.9%	209 (75.7%)	74.1–77.3%			
6	46 (17.5%)	16.1–18.9%	217 (82.5%)	81.1–83.9%			
7	43 (17.4%)	16.0–18.8%	204 (82.6%)	81.2–84.0%			
8	50 (18.3%)	16.9–19.7%	223 (81.7%)	80.3–83.1%			
9	37 (13.5%)	12.2–14.8%	237 (86.5%)	85.2–87.8%			
10	45 (15.1%)	13.8–16.4%	253 (84.9%)	83.6–86.2%			
Region of England					43.962	0.130	<0.001
North East	21 (19.3%)	17.8–20.8%	88 (80.7%)	79.2–82.2%			
North West	65 (26.3%)	24.7–27.9%	182 (73.7%)	72.1–75.3%			
Yorkshire and the Humber	117 (29.7%)	28.0–31.4%	277 (70.3%)	68.6–72.0%			
East Midlands	60 (31.3%)	29.6–33.0%	132 (68.8%)	67.1–70.5%			
West Midlands	72 (19.6%)	18.1–21.1%	296 (80.4%)	78.9–81.9%			
East of England	87 (20.5%)	19.0–22.0%	336 (79.4%)	77.9–80.9%			
London	33 (12.4%)	11.2–13.6%	233 (87.6%)	86.4–88.8%			
South East	118 (22.2%)	20.6–23.8%	413 (77.8%)	76.2–79.4%			
South West	34 (16.3%)	14.9–17.7%	174 (83.7%)	82.3–85.1%			
Free school meal eligibility					18.2	−0.082	<0.001
No	349 (19.7%)	15.5–23.9%	1425 (80.3%)	78.2–82.4%			
Yes	258 (26.8%)	21.4–32.2%	706 (73.2%)	70.0–76.5%			
Ethnicity					15.16	0.075	0.004
White	560 (22.9%)	21.3–24.5%	1885 (77.1%)	75.5–78.7%			
Asian	15 (12.6%)	11.4–13.8%	104 (87.4%)	86.2–88.6%			
Black	<10		<24				
Mixed	22 (16.7%)	15.3–18.1%	110 (83.3%)	81.9–84.7%			
Any other ethnic group	<10		<18				

CI, confidence interval; EHCP, education, health and care plan; IMD, index of multiple deprivation; PHI, equivalent to correlation coefficient.

### Region of domicile

**Figure 2:**
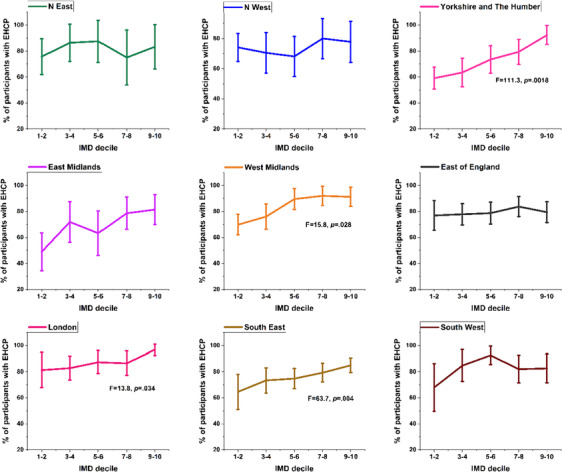
Proportion of participants with education, health and care plan (EHCP) within the index of multiple deprivation (IMD) quintiles in the nine regions of England, with 95% confidence interval error bars.

The number of participants from each region of England was generally proportionate to the region's population size, with some small variation (Table 1). In contrast, the proportions of participants who had been granted an EHCP varied markedly between regions. London had the highest proportion (nearly 88%), whereas in the East Midlands, the proportion was less than 70% (*χ* ^2^ = 43.962, *P* < 0.001; Table 5). The proportion of participants with an EHCP within each IMD decile in the nine regions of England is presented in Fig. 2. In some regions, pupils in more advantaged deciles were much more likely to have obtained an EHCP than those in the lowest decile: Yorkshire and the Humber (*F* = 111.3, *P* = 0.0018), West Midlands (*F* = 15.8, *P* = 0.028), London (*F* = 13.8, *P* = 0.034) and South East (*F* = 63.7, *P* = 0.004). London schools had consistently higher proportions of participants with an EHCP within all IMD deciles (between 80% and 97% from the lowest to highest IMD deciles; Fig. 2).

### Eligibility for free school meal

In this cohort, a significantly higher proportion of participants (35.2%; Table 1) were eligible for FSM compared with the national figure (22.5%) in 2022 (UK Government 2022c). Proportionately more participants who were eligible for FSM lived in more deprived areas (*χ* ^2^ = 334.8, *P* < 0.001; Table S2). Among pupils with FSM eligibility, a significantly lower proportion (73%) had been granted an EHCP, compared with 80% of those without eligibility for FSM (*χ* ^2^ = 18.2, *P* < 0.001; Table 5).

### Ethnicity

Children of Asian ethnic origin were relatively more likely to have been granted an EHCP than White participants. Comparative figures were as follows: Asian (87%), mixed race (83%) and White (77%), respectively (*χ* ^2^ = 15.16, *P* = 0.004; Table 5).

### Summary

A stepwise logistic regression examined the association between obtaining an EHCP (dependent variable) and an IMD decile (independent variable), covarying for region of England, FSM eligibility, ethnicity and sex. The univariate association between IMD and the odds of obtaining an EHCP showed that participants living in more advantaged IMD deciles were between 1.5 and nearly 4 times higher odds to have been granted an EHCP than those living in the most deprived area (i.e. IMD decile 1; see step 1 in Table 6). In the fully adjusted model, the odds of receiving an EHCP by IMD decile remained remarkably similar [odds ratio (OR) ranged from 95% confidence interval (CI) = 1.14–2.44, *P* < 0.008, to 95% CI = 2.23–5.37, *P <* 0.001; step 5 in Table 6]. Covariates, region of England and ethnicity, were also significant predictors of receiving an EHCP. Those living in regions outside of London were at significantly lower odds of receiving an EHCP than those living in London: North West (OR = 0.53, 95% CI = 0.33–0.86, *P* < 0.010), Yorkshire and the Humber (OR = 0.45, 95% CI = 0.29–0.69, *P* < 0.001), East Midlands (OR = 0.37, 95% CI = 0.23–0.60, *P* < 0.001), East of England (OR = 0.61, 95% CI = 0.39–0.95, *P* = 0.030) and South East (OR = 0.52, 95% CI = 0.34–0.79, *P* < 0.002). In other words, pupils living in London were two to three times more likely to have been granted an EHCP than those living in other regions of England. FSM eligibility and sex were not significant predictors, but, compared with White participants, children from Asian families had greater odds of receiving an EHCP (95% CI = 1.20–3.69, *P* = 0.010).

**Table 6:** Stepwise logistic regressions to present the odds of EHCP having been granted based on families' IMD decile, region of domicile, the child's eligibility for free school meals, ethnic origin and sex (*N* = 2738)

Stepwise logistic regression	Step 1		Step 2		Step 3		Step 4		Step 5	
	OR (95% CI)	*P*	OR (95% CI)	*P*	OR (95% CI)	*P*	OR (95% CI)	*P*	OR (95% CI)	*P*
IMD decile (decile 1 as ref)										
2	1.53 (1.08–2.18)	0.017*	1.39 (0.97–1.98)	0.071	1.36 (0.95–1.94)	0.092	1.33 (0.93–1.90)	0.119	1.34 (0.94–1.92)	0.107
3	2.04 (1.41–2.96)	<0.001***	1.90 (1.30–2.77)	<0.001***	1.87 (1.28–2.72)	<0.001***	1.86 (1.27–2.72)	<0.001***	1.86 (1.27–2.71)	<0.001***
4	1.56 (1.09–2.25)	0.015*	1.40 (0.97–2.03)	0.076	1.35 (0.93–1.96)	0.119	1.34 (0.92–1.96)	0.123	1.35 (0.93–1.97)	0.116
5	1.83 (1.28–2.62)	<0.001***	1.73 (1.19–2.50)	0.004**	1.64 (1.13–2.39)	0.010*	1.65 (1.13–2.41)	0.009**	1.67 (1.14–2.44)	0.008**
6	2.77 (1.86–4.10)	<0.001***	2.55 (1.70–3.82)	<0.001***	2.40 (1.59–3.62)	<0.001***	2.43 (1.61–3.68)	<0.001***	2.47 (1.63–3.73)	<0.001***
7	2.78 (1.86–4.16)	<0.001***	2.64 (1.74–3.99)	<0.001***	2.48 (1.63–3.78)	<0.001***	2.57 (1.68–3.92)	<0.001***	2.62 (1.72–4.00)	<0.001***
8	2.62 (1.78–4.85)	<0.001***	2.50 (1.69–3.71)	<0.001***	2.33 (1.56–3.49)	<0.001***	2.38 (1.59–3.57)	<0.001***	2.40 (1.60–3.61)	<0.001***
9	3.77 (2.47–5.70)	<0.001***	3.53 (2.30–5.41)	<0.001***	3.28 (2.12–5.07)	<0.001***	3.41 (2.20–5.30)	<0.001***	3.46 (2.23–5.37)	<0.001***
10	3.30 (2.22–4.89)	<0.001***	3.12 (2.08–4.70)	<0.001***	2.90 (1.91–4.41)	<0.001***	2.98 (1.96–4.54)	<0.001***	3.04 (2.00–4.64)	<0.001***
Region of England (London as ref)										
North East	—	—	0.73 (0.39–1.34)	0.303	0.73 (0.40–1.34)	0.308	0.81 (0.44–1.50)	0.50	0.82 (0.44–1.51)	0.517
North West	—	—	0.48 (0.30–0.77)	0.002**	0.48 (0.30–0.76)	0.002**	0.53 (0.33–0.85)	0.008**	0.53 (0.33–0.86)	0.010*
Yorkshire and the Humber	—	—	0.40 (0.26–0.62)	<0.001***	0.40 (0.26–0.62)	<0.001***	0.45 (0.29–0.69)	<0.001***	0.45 (0.29–0.69)	<0.001***
East Midland	—	—	0.33 (0.21–0.54)	<0.001***	0.34 (0.21–0.54)	<0.001***	0.37 (0.23–0.60)	<0.001***	0.37 (0.23–0.60)	<0.001***
West Midland	—	—	0.71 (0.45–1.11)	0.136	0.70 (0.45–1.11)	0.130	0.77 (0.48–1.21)	0.256	0.77 (0.49–1.23)	0.274
East of England	—	—	0.55 (0.36–0.85)	0.008**	0.55 (0.45–1.11)	0.008**	0.61 (0.39–0.95)	0.029*	0.61 (0.39–0.95)	0.030*
South East	—	—	0.47 (0.31–0.71)	<0.001***	0.46 (0.36–0.85)	<0.001***	0.51 (0.33–0.78)	0.002**	0.52 (0.34–0.79)	0.002**
South West	—	—	0.72 (0.43–1.22)	0.224	0.72 (0.43–1.22)	0.223	0.82 (0.48–1.41)	0.449	0.82 (0.48–1.39)	0.456
FSM eligibility (yes as ref)										
No	—	—	—	—	1.17 (0.96–1.43)	0.130	1.15 (0.94–1.41)	0.172	1.16 (0.95–1.43)	0.142
Ethnicity (White as ref)										
Asian	—	—	—	—	—	—	2.10 (1.20–3.69)	0.010*	2.10 (1.20–3.69)	0.010*
Black	—	—	—	—	—	—	2.26 (0.65–7.83)	0.200	2.39 (0.69–8.32)	0.171
Mixed	—	—	—	—	—	—	1.46 (0.91–2.36)	0.121	1.45 (0.90–2.34)	0.130
Any other ethnic group	—	—	—	—	—	—	0.45 (0.17–1.21)	0.114	0.45 (0.17–1.20)	0.110
Sex (male as ref)										
Female	—	—	—	—	—	—	—	—	1.2 (1.0–1.44)	0.05

* *P* < 0.05.

** *P* < 0.01.

*** *P* < 0.001.

CI, confidence interval; EHCP, education, health and care plan; FSM, free school meal; IMD, index of multiple deprivation; OR, odds ratio; ref, reference.

## Discussion

In this nationwide IMAGINE study of CYP with IDDs of genetic aetiology, we investigated the factors that influenced the awarding of support from EHCPs. In the state education system, all participants consistently achieved below the national standard of educational progress as measured by statutory assessments. The odds of receiving support from an EHCP were influenced by the family's socioeconomic status (as measured by the IMD score), region of domicile and ethnicity, but not by the child's sex or by the degree of an associated emotional or behavioural disorder.

On average, all participants had high or very high degrees of difficulty compared with children without IDD, a finding that is consistent with previous research (Emerson & Hatton 2007; Hughes‐McCormack *et al*. 2017; Adlington *et al*. 2019). Children with and without an EHCP had similar degrees of emotional and behavioural maladjustment. Because placement in special schools for children with IDD would require an EHCP, those without an EHCP all attended mainstream establishments (a few with specialised units), where they may have been at greater risk especially during adolescence. SEND pupils are eight to nine times more likely to be permanently excluded than their typical peers (Davis 2012; Parker *et al*. 2016). Pupils with IDD are some of the most socially excluded and bullied pupils in the school system (Green *et al*. 2010; Naylor *et al*. 2012; Maguire *et al*. 2018).

There were no significant differences in the EYFSP scores at the age of 4–5 years between the cohort participants who subsequently were granted an EHCP and those who were not. Reports suggest that the EYFSP score could be used as a tool to identify children who are at increased risk of needing SEN support (Wright *et al*. 2019; Atkinson *et al*. 2022; Wood *et al*. 2024). The EYFSP score assesses a range of abilities at school entry including academic, language, socio‐emotional and motor skills (Atkinson *et al*. 2022). At that time, during the reception year, both cohort groups showed very similar degrees of developmental delay (Fig. 1a). Although the cohort participants without an EHCP tended to obtain higher statutory assessment scores than those with an EHCP at later academic stages, they had persistently poor attainment compared with children from the general population (Fig. 1b–d), indicating that both groups of pupils needed similar additional educational support. A recent report shows that 23.1% of parents of children with a genetic disorder associated with neurodevelopmental delay feel that their child's school does not provide their child with the right educational support (Mon‐Williams & Wood 2023).

The current UK government has aimed to provide additional funding for schools in England with the highest concentration of disadvantaged children in order to raise attainment levels (Anders & Henderson 2019). Nevertheless, proportionately more cohort children who lived in the most deprived IMD deciles lacked an EHCP compared with those in the least deprived deciles. Higher rates of IDD have been found among children living in more deprived areas (Laxton *et al*. 2024). These children are at greater risk of poor developmental outcomes compared with children in less deprived areas (Siddiqua *et al*. 2020) and are arguably more in need of EHCP support. Limited funding and subsequent limited availability (or lack) of services in deprived areas may explain why families living in the most deprived areas access fewer services (Laxton *et al*. 2024).

We also identified substantial variations in EHCP provision between English regional education authorities (Marsh & Howatson 2020), and mainstream schools in England are not motivated to be inclusive of pupils with SEND (House of Commons, U.K. 2020). Participants living in London had a notably higher chance of obtaining an EHCP compared with other regions of England. The most advantaged families in London were nearly 100% successful in obtaining an EHCP for their child, whereas in the East Midlands and Yorkshire and the Humber, the most disadvantaged families (whose children had similar disabilities) were only 70% successful. Regional differences in EHCP provisions are likely due to unequal funding across the regions of England; on average, pupils in London received 9.7% more funding than those in the North of England (Mon‐Williams & Wood 2023). There has been remarkably slow progress in providing adequately for pupils with greater needs since the SEND reforms in 2014 (Hutchinson *et al*. 2020). Children without an EHCP who attended mainstream state schools received no additional funding, putting them at a substantial disadvantage (Hutchinson *et al*. 2020). This is also a worldwide phenomenon, as one study shows higher prevalence rates of IDD in the lower socio‐demographic regions globally and concludes that relative inequalities continue to rise with lower socio‐demographic regions needing more comprehensive support services (Nair *et al*. 2022).

The current study has some limitations. The IMAGINE cohort did not contain proportionately equivalent participants from minority ethnic groups, particularly Black and Asian children, compared with the national census data. On the other hand, those Asian participants for whom we have DfE data were significantly more likely to have received an EHCP compared with White participants. Others have commented that by the end of secondary education, some minority ethnic groups have relatively greater attainments than the majority. Chinese pupils are 2 years ahead and Indian pupils are 15 months ahead of the general population of children (Hutchinson *et al*. 2020). The children of some minority groups are more likely to have taken higher‐level public examinations and are more likely to be university educated than White British families (Crawford & Greaves 2015). This disparity could reflect differential parental attitudes; ethnic minority pupils report their families have particularly high expectations of their educational progress (Conner *et al*. 2004). Such cultural attitudes suggest Asian families could be pursuing an EHCP for their disabled child more vigorously than others.

Another limitation is that we have not received educational attainment data for every IMAGINE cohort participant. Some state schools use an educational evaluation system for SEND pupils that differs from the national standardised scoring system, beyond the EYFSP (Wearmouth 2022). Another limitation concerns the necessity of selecting a single primary SEN type to represent a single pupil. In practice, that pupil could have been assigned multiple primary SEN types during their education, in different academic years. Finally, although we have estimates of the child's mental health from (SDQ) assessments based on parental or primary carer reports, teacher assessments were not available.

## Conclusions

This study has documented a range of demographic and regional inequalities that influence the chance that young people who have IDD associated with genetic disorders are granted EHCP to support their educational progress in English state schools. Whilst pupils who live in the most deprived areas (by IMD decile) are relatively less likely to have support from an EHCP than those in the least deprived deciles, wherever they live, there are also marked regional anomalies. In London, nearly 100% of participants from the most advantaged socioeconomic stratum had been awarded such support. Equity of access to education means that differences in students' outcomes, such as academic performance, social and emotional well‐being, and post‐secondary educational attainment, should not depend on their socioeconomic background (Organisation for Economic Co‐operation and Development 2018).

## Source of Funding

This research project was funded by the Medical Research Council, London, UK. No external funding was received for the research reported in the paper.

## Conflict of Interest

The authors report no conflicts of interest.

## Ethics Approval and Consents

The study was approved by the London Queen Square Research Ethics Committee in the UK (14/LO/1069). Written or online consent was obtained from the parents/caregivers of children under the age of 16 or from the consultees or participants over the age of 16 who took part in this study.

## Supporting information


**Table S1.** The frequency and percentage of the secondary special educational need types of the cohort participants with and without Education Health Care Plan.
**Note S1.** More information about the SDQ measures.
**Note S2.** The use of the term “EHCP” in the manuscript.
**Table S2.** The Free School Meal eligibility of the cohort participants living in the ten Index of Multiple Deprivation deciles.
**Appendix.** IMAGINE ID Consortium members.

## Data Availability

The data from the UK Department for Education (DfE) that supports the findings in this paper are not available for data sharing according to the guidelines of DfE and the Office for National Statistics (ONS). Other research data from this study are available from the authors upon application. This work contains statistical data from ONS, which is Crown copyright. The use of the ONS statistical data in this work does not imply the endorsement of the ONS in relation to the interpretation or analysis of the statistical data. This work uses research datasets, which may not exactly reproduce National Statistics aggregates.
