# Supplementary material for: The inequity of education, health and care plan provision for children and young people with intellectual and developmental disabilities
Source: J Intellect Disabil Res. 2024 May 22;68(10):1167–83. doi: 10.1111/jir.13139 (PMC11753480; doi:10.1111/jir.13139)
Supplement: Supplementary file 1 — Table S1. The frequency and percentage of the secondary special educational need types of the cohort participants with and without Education Health Care Plan. Note S1. More information about the SDQ measures. Note S2. The use of the term “EHCP” in the manuscript. Table S2. The Free School Meal eligibility of the cohort participants living in the ten Index of Multiple Deprivation deciles. Appendix. IMAGINE ID Consortium members. [file JIR-68-1167-s001.docx]

**Supplementary Materials**

**The inequity of education health care plan provision for children and young people with intellectual and developmental disabilities**

Irene O Lee^1^, Jeanne Wolstencroft^1^, Harriet Housby^1^, Marianne B M van den Bree^2^, Samuel J R A Chawner^2^, Jeremy Hall^2^, IMAGINE ID Consortium and David H Skuse^1^

^1^ Behavioural and Brain Sciences Unit, Population Policy and Practice Programme, Great Ormond Street Institute of Child Health, University College London, London, UK.

^2^ Centre for Neuropsychiatric Genetics and Genomics, Division of Psychological Medicine and Clinical Neurosciences, Cardiff University, Cardiff, UK.

^3^ IMAGINE ID Consortium, see Appendix.

**Contents**

[**Supplementary Table S1**. The frequency and percentage of the secondary special educational need types of the cohort participants with and without Education Health Care Plan. 1](#_Toc164258204)

[**Supplementary Note S1.** More information about the SDQ measures. 2](#_Toc164258205)

[**Supplementary Note S2.** The use of the term “EHCP” in the manuscript. 4](#_Toc164258206)

[**Supplementary Table S2.** The Free School Meal eligibility of the cohort participants living in the ten Index of Multiple Deprivation deciles. 5](#_Toc164258207)

[**References** 6](#_Toc164258208)

[**Appendix. IMAGINE ID Consortium members** 7](#_Toc164258209)

**Supplementary Table S1**. The frequency and percentage of the secondary special educational need types of the cohort participants with and without Education Health Care Plan.

| Secondary SEN Type | Frequency (%  of total count) | No EHCP (% of  SEN subtype) | Have EHCP (%  of SEN subtype) |
| --- | --- | --- | --- |
| PMLD | 56 (2.0%) | <10 | <66 (>96%) |
| Severe LD | 90 (3.3%) | <10 | <100 (>98%) |
| Moderate LD | 282 (10.3%) | 54 (19.1%) | 228 (80.9%) |
| Specific LD | 170 (6.2%) | 35 (20.6%) | 135 (79.4%) |
| SLCN | 582 (21.3%) | 81 (13.9%) | 501 (86.1%) |
| ASD | 242 (8.8%) | 26 (10.7%) | 216 (89.3%) |
| SEMH + BESD | 179 (6.5%) | 60 (33.5%) | 119 (66.5%) |
| MSI + HI + VI | 98 (6.9%) | 19 (19.4%) | 79 (80.6%) |
| PD | 188 (4.4%) | 30 (16%) | 158 (84%) |
| Others | 120 (4.4%) | 37 (30.8%) | 83 (69.2%) |
| Missing Data | 731 (26.7%) | --- | --- |

ASD=Autistic Spectrum Disorder; BESD=Behavioural Emotional Social Difficulty; EHCP=Education Health Care Plan; HI=Hearing Impairment; LD=Learning Difficulty; MSI=Multi-Sensory Impairment; PD=Physical Disability; PMLD=Profound and Multiple Learning Difficulty; Others= Other difficulties/disabilities; SEMH=Social, Emotional and Mental Health; SEN=Special Educational Need; SLCN=Speech, Language and Communications needs; VI =Visual Impairment. <10=count less than ten cannot be presented according to the ONS guidelines.

# **Supplementary Note S1.** More information about the SDQ measures.

The Strengths and Difficulties Questionnaire (SDQ)(Goodman, 1997) is a brief behavioural screening questionnaire about 2–17 year olds, and is a globally recognised instrument for assessing the mental health status for children and young people (Terapia, 2020). SDQ scoring provides an overview to evaluate children and young people’s emotional and behavioural adjustment in dimensional terms (Goodman & Goodman, 2009; Murray et al., 2020).

There are 25 items in the SDQ which comprises of 5 scales in 5 items each that measure: emotional symptoms; conduct problems; hyperactivity, impulsivity, and inattention difficulties; peer relationship problems; and prosocial behaviour. The first four of these scales are combined to make a total difficulties score. Higher scores are indicative of a greater mental health difficulty and scores above 90th percentile indicate a high probability of a diagnosable psychiatric disorder; whereas a lower score on the prosocial behaviour implies greater difficulties (Goodman & Goodman, 2009; Goodman, 2001). The SDQ has been used as a standardised assessment to evaluate emotional and behavioural difficulties of children and adolescents with intellectual and developmental disabilities (Murray et al., 2020).

The SDQ scores were categorised into four bands based on a general population UK survey: 80% of UK children score in a “Close to Average” range regarded as ‘normal’, 10% score “Slightly Raised” range as ‘borderline’, and 10% score “High” or “Very High” range regarded as ‘abnormal’ (Terapia, 2020).

Categorisation bands for SDQ scores are listed in the following table to identify the correct classification for each score.

For Parent/Carer completed SDQ:

| **Classification for each SDQ subscale score** | **Close to**  **Average**  (80% pop) | **Slightly raised**  **(/lowered)**  (10% pop) | **High**  **(/Low)**  (5% pop | **Very high**  **(/very low)**  (5% pop) |
| --- | --- | --- | --- | --- |
| Emotional problems score | 0-3 | 4 | 5-6 | 7-10 |
| Conduct problems score | 0-2 | 3 | 4-5 | 6-10 |
| Hyperactivity score | 0-5 | 6-7 | 8 | 9-10 |
| Peer problems score | 0-2 | 3 | 4 | 5-10 |
| Prosocial score | 8-10 | 7 | 6 | 0-5 |
| **Total difficulties score** | 0-13 | 14-16 | 17-19 | 20-40 |

Note: pop=population.

For further information regarding the scoring process, please visit: <https://sdqscore.org/>.

# **Supplementary Note S2.** The use of the term “EHCP” in the manuscript.

Some participants had both SEN Statement and then had EHCP during the time they were in the education system (between reception and post 19 education), whereas some had only EHCP if they started their education after 2014 when EHCP was first introduced. The breakdown are as follows:

Just had SEN Statement= 154

Just had EHCP=945

Had both SEN Statement and EHCP= 1032

The term “SEN statement” has been discontinued since 2014. For clarity we used the term “EHCP” as it is a current term to describe the provisions for their additional needs in the manuscript.

**Supplementary Table S2.** The Free School Meal eligibility of the cohort participants living in the ten Index of Multiple Deprivation deciles.

| IMD decile | FSM Eligibility | | χ2 | p-value |
| --- | --- | --- | --- | --- |
|  | **No** | **Yes** | 334.8 | <.001 |
| 1 (most deprived) | 103 (34.3%) | 199 (65.7%) |  |  |
| 2 | 137 (48.6%) | 145 (51.4%) |  |  |
| 3 | 124 (46.6%) | 145 (53.9%) |  |  |
| 4 | 150 (59.3%) | 103 (40.7%) |  |  |
| 5 | 187 (67.8%) | 89 (32.3%) |  |  |
| 6 | 194 (73.8%) | 69 (26.2%) |  |  |
| 7 | 182 (73.7%) | 65 (26.3%) |  |  |
| 8 | 218 (79.9%) | 55 (20.1%) |  |  |
| 9 | 228 (83.2%) | 46 (16.8%) |  |  |
| 10 (least deprived) | 250 (83.9%) | 48 (16.1%) |  |  |

FSM=Free School Meal; IMD=Index of Multiple Deprivation.

# **References**

Goodman, A., & Goodman, R. (2009). Strengths and Difficulties Questionnaire as a Dimensional Measure of Child Mental Health. *Journal of the American Academy of Child and Adolescent Psychiatry*, *48*(4), 400-403. <https://sdqscore.org/>

Goodman, R. (1997). The Strengths and Difficulties Questionnaire: a research note. *J Child Psychol Psychiatry*, *38*, 581–586. <https://doi.org/https://acamh.onlinelibrary.wiley.com/doi/epdf/10.1111/j.1469-7610.1997.tb01545.x>

Goodman, R. (2001). Psychometric properties of the strengths and difficulties questionnaire. *J Am Acad Child Adolesc Psychiatry*, *40*, 1337-1345.

Murray, C. A., Hastings, R. P., & Totsika, V. (2020). Clinical utility of the parentreported Strengths and Difficulties Questionnaire as a screen for emotional and behavioural difficulties in children and adolescents with intellectual disability. *Br J Psychiatry*, *218*, 323-325.

Terapia. (2020). *Scoring Strengths and Difficulties Questionnaire for age 4-17 or 18+*. <https://terapia.co.uk/wp-content/uploads/2020/05/SDQ-scoring_Instructions_4-18-years.pdf>

# **Appendix. IMAGINE ID Consortium members**

| **Surname** | **Initials** | **First Name** | **Title** | **Institution** |
| --- | --- | --- | --- | --- |
| Housby | H | Harriet | Ms | Great Ormond Street Institute of Child Health, University College London, UK |
| Lee | I | Irene | Mrs | Great Ormond Street Institute of Child Health, University College London, UK |
| Skuse | D | David | Professor | Great Ormond Street Institute of Child Health, University College London, UK |
| Wolstencroft | J | Jeanne | Dr | Great Ormond Street Institute of Child Health, University College London, UK |
| Mandy | W | William | Dr | Division of Psychology & Language Sciences, University College London, UK |
| Denaxas | S | Spiros | Dr | Institute of Health Informatics, University College London, London, UK |
| van den Bree | MBM | Marianne | Professor | Centre for Neuropsychiatric Genetics and Genomics, Division of Psychological Medicine and Clinical Neurosciences, Cardiff University, UK |
| Chawner | SJRA | Samuel | Dr | Centre for Neuropsychiatric Genetics and Genomics, Division of Psychological Medicine and Clinical Neurosciences, Cardiff University, UK |
| Hall | J | Jeremy | Professor | Centre for Neuropsychiatric Genetics and Genomics, Division of Psychological Medicine and Clinical Neurosciences, Cardiff University, UK |
| Holmans | P | Peter | Professor | Centre for Neuropsychiatric Genetics and Genomics, Division of Psychological Medicine and Clinical Neurosciences, Cardiff University, UK |
| Hope-Bell | J | Josh | Dr | Centre for Neuropsychiatric Genetics and Genomics, Division of Psychological Medicine and Clinical Neurosciences, Cardiff University, UK |
| Le Roux | D | Danielle | Ms | Centre for Neuropsychiatric Genetics and Genomics, Division of Psychological Medicine and Clinical Neurosciences, Cardiff University, UK |
| Morrin | S | Sally | Ms | Centre for Neuropsychiatric Genetics and Genomics, Division of Psychological Medicine and Clinical Neurosciences, Cardiff University, UK |
| Owen | MJ | Michael | Professor Sir | Centre for Neuropsychiatric Genetics and Genomics, Division of Psychological Medicine and Clinical Neurosciences, Cardiff University, UK |
| Sivakumar | S | Shreeya | Ms | Centre for Neuropsychiatric Genetics and Genomics, Division of Psychological Medicine and Clinical Neurosciences, Cardiff University, UK |
| Baker | K | Kate | Dr | Department of Medical Genetics, University of Cambridge, UK |
| Raymond | FLF | Lucy | Professor | Department of Medical Genetics, University of Cambridge, UK |
